# Cadmium exposure is associated with increased transcript abundance of multiple heavy metal associated transporter genes in roots of hemp (*Cannabis sativa* L.)

**DOI:** 10.3389/fpls.2023.1183249

**Published:** 2023-05-30

**Authors:** Amanda O. Marabesi, Savithri U. Nambeesan, Marc W. van Iersel, Jason T. Lessl, Timothy W. Coolong

**Affiliations:** ^1^Department of Horticulture, University of Georgia, Athens, GA, United States; ^2^Agricultural and Environmental Services Lab, University of Georgia, Athens, GA, United States

**Keywords:** accumulation, cadmium, cannabinoids, *Cannabis sativa*, transporter genes, heavy metals

## Abstract

Industrial hemp (*Cannabis sativa* L.) has demonstrated promise for phytoremediation due to an extensive root system, large biomass, and ability to survive under relatively high levels of heavy metals. However, little research has been conducted to determine the impact of heavy metal uptake in hemp grown for medicinal use. This study evaluated the potential for cadmium (Cd) uptake and its impact on growth, physiological responses, and transcript expression of metal transporter genes in a hemp variety grown for flower production. The cultivar ‘Purple Tiger’ was exposed to 0, 2.5, 10, and 25 mg·L^-1^ Cd in a greenhouse hydroponic study in two independent experiments. Plants exposed to 25 mg·L^-1^ Cd displayed stunted plant growth characteristics, reduced photochemical efficiency, and premature senescence suggesting Cd toxicity. At the two lower concentrations of Cd (2.5 and 10 mg·L^-1^ Cd), plant height, biomass, and photochemical efficiency were not affected, with chlorophyll content index (CCI) being slightly lower at 10 mg·L^-1^ Cd, compared to 2.5 mg·L^-1^ Cd. There were no consistent differences between the two experiments in total cannabidiol (CDB) and tetrahydrocannabinol (THC) concentrations in flower tissues at 2.5 and 10 mg·L^-1^ Cd, compared to the control treatment. Root tissue accumulated the highest amount of Cd compared to other tissues for all the Cd treatments, suggesting preferential root sequestration of this heavy metal in hemp. Transcript abundance analysis of heavy metal-associated (HMA) transporter genes suggested that all seven members of this gene family are expressed in hemp, albeit with higher expression in the roots than in the leaves. In roots, *CsHMA3* was up-regulated at 45 and 68 d after treatment (DAT), and *CsHMA1*, *CsHMA*4, and *CsHMA*5 were upregulated only under long term Cd stress at 68 DAT, at 10 mg·L^-1^ Cd. Results suggest that expression of multiple HMA transporter genes in the root tissue may be upregulated in hemp exposed to 10 mg·L^-1^ Cd in a nutrient solution. These transporters could be involved in Cd uptake in the roots *via* regulating its transport and sequestration, and xylem loading for long distance transport of Cd to shoot, leaf, and flower tissues.

## 1 Introduction

Hemp (*Cannabis sativa* L.) has been cultivated for centuries for feed, fiber, and medicinal purposes (Ranalli, 1999). In the United States (U.S.), production has been largely prohibited until the passage of the 2018 Agriculture Improvement Act (Farm Bill), which allowed for legal cultivation of industrial hemp [US Department of Agriculture, 2019]. Industrial hemp is defined as *C. sativa* with a total delta-9 tetrahydrocannabinol (THC) concentration of less than 0.3% on a dry-weight basis. *C. sativa* plants with delta-9 THC concentrations above 0.3% are classified as marijuana and are federally prohibited in the U.S. Tetrahydrocannabinol and cannabidiol (CBD) are two of the more than 100 cannabinoids found in *C. sativa* plants, many of which have therapeutic uses such as the treatment of anxiety and seizures (ElSohly and Gul, 2014; National Academies of Sciences, Engineering, and Medicine, 2017; US Department of Agriculture, 2019). Although legalized by the U.S. federal government, there are significant regulations for industrial hemp production that must be followed to stay in compliance with federal law. Further, processors often impose strict quality control standards for their products as they may be consumed as dietary supplements. Heavy metal accumulation in hemp biomass is an area of concern for processors and many have attempted to implement standards regarding maximum allowable levels of metals.

Previously, human exposure to cadmium (Cd), has been associated with smoking contaminated tobacco (*Nicotiana tabacum* L.), particularly counterfeit cigarettes sold in developing markets (Akesson & Chaney, 2019). Cadmium accumulation in smokable plants, such as tobacco and hemp, is concerning for human health due to the harmful effects of Cd inhalation, which has been associated with renal tubular dysfunction, osteomalacia, lung disease, and it is classified as a Group 1 carcinogen (Gauvin et al., 2018; Akesson & Chaney, 2019; Ismael et al., 2019). Nonetheless, consumers are less likely to perceive the harms of *C. sativa* smoke to the same extent as those caused by smoking tobacco (Gauvin et al., 2018). Cadmium is a naturally occurring element and is found in trace amounts in the environment, including soil. Cadmium may be introduced to agricultural soils *via* contaminated manure and biosolids, phosphate fertilizers, mine waste, atmospheric deposition from smelter emissions, and industrial waste (Chaney, 2010; Sebastian et al., 2019).

Studies have documented the ability of hemp to survive on soils with relatively high levels of heavy metals, including Cd, copper (Cu), nickel (Ni), lead (Pb), zinc (Zn), mercury (Hg), and chromium (Cr) (Linger et al., 2002; Citterio et al., 2003; Angelova et al., 2004; Linger et al., 2005; Shi & Cai, 2009; Shi & Cai, 2010; Shi et al., 2012; Ahmad et al., 2015; Galic et al., 2019; Husain et al., 2019). Furthermore, hemp has been suggested to have the potential to remove Cd from contaminated soils for phytoremediation purposes. Hemp shares some traits with species of plants that can establish themselves in otherwise phytotoxic environments, such as an extensive and deep root system, efficient translocation of contaminants to the shoots, rapid life cycle, and high biomass production (Citterio et al., 2003; Nesler & Furini, 2012).

Numerous plant species have developed mechanisms to cope with exposure to high levels of heavy metals (Eutropio et al., 2016). The first step in Cd accumulation in plants is the uptake of Cd ions by the roots. The rhizosphere is critical in this process as it can provide an appropriate environment for the existence of bioavailable forms of metals, in terms of pH, redox potential, and presence of microorganisms (Chaney, 2010; Lux et al., 2011; Ismael et al., 2019; Sebastian et al., 2019). Subsequently, metal ions may enter roots through protein transporters and travel *via* apoplastic or symplastic pathways. Once in root cells, metal cations may be chelated with phytochelatins, metallothioneins, organic acids, and amino acids and stored in the vacuoles in a chelated form, while also being loaded into the xylem where they are translocated to the shoots and leaves. Cadmium accumulation in hemp has been reported to be greater in roots when compared to other tissues, though translocation of Cd to aboveground portions of plants is still significant (Citterio et al., 2003; Linger et al., 2005; Shi & Cai, 2009; Shi et al., 2012; Ahmad et al., 2015; Galic et al., 2019).

Metal transporters mediate Cd movement within the plant. These proteins primarily transport divalent cations such as Cu, Zn, calcium (Ca), magnesium (Mg), and iron (Fe), which are essential nutrients for plants. When Cd is present in the soil solution, it competes with these essential nutrients for transport, and may be colloquially referred to as an “opportunistic hitchhiker” (Shah et al., 2019). Several transport protein families are involved in the uptake and movement of heavy metals in plants (Lux et al., 2011; Ismael et al., 2019; Sebastian et al., 2019). Transporting ATPases such as heavy metal ATPase 2 (*AtHMA2*) and heavy metal ATPase 4 (*AtHMA4*) have been reported to play an important role on Cd uptake and translocation in *Arabidopsis thaliana* (L.) Heynh (Wong & Cobbett, 2009), while *AtHMA3* (Morel et al., 2009; Ismael et al., 2019) and its orthologues *OsHMA3* in *Oriza sativa* L. are involved in vacuolar loading of Cd (Miyadate et al., 2011; Ismael et al., 2019).

Prior research has largely focused on the survival and accumulation of Cd in fiber hemp varieties grown for phytoremediation purposes. However, much of the hemp intended for human consumption is specifically grown for floral production, which is utilized for cannabinoid extraction or directly for smoking. Therefore, there is interest in determining the potential for Cd contamination in flower tissue. There is a lack of research-based information on heavy metal accumulation in hemp grown for the medicinal market and the relationship between Cd stress and hemp growth and development (Ahmad et al., 2015; Yin et al., 2022). In this study we evaluated the potential for Cd accumulation and its impact on growth and physiological responses related to photochemical efficiency and chlorophyll content, and transcript abundance of metal transporter genes in an industrial hemp variety grown specifically for flower (cannabinoid) production.

## 2 Materials and methods

### 2.1 Experimental setting

Two independent greenhouse experiments utilizing a deep-water culture hydroponic system were conducted from Dec. 2020 through Mar. 2021 and Feb. 2021 through May 2021. Industrial hemp ‘Purple Tiger’, developed for flower production (The Hemp Mine, Fair Play, SC, USA), was propagated *via* rooted cuttings. This cultivar was chosen due to its long photoperiod requirements for vegetative growth (≥ 17 h) and ability to accumulate CBD in the flower tissue. Cuttings were taken from female plants in the active vegetative growth phase containing three nodes each. Cuttings were dipped in a commercial rooting gel (0.31% indole butyric acid; CLONEX, Growth Technology Ltd., Somerset, UK) and placed into engineered foam cubes (3.33 cm L x 2.54 cm W x 3.81 cm D; Oasis Grower Solutions, Kent, OH, USA) for rooting. Foam cubes were placed in plastic trays located on a heat mat set at 24°C under a mist system, which applied water approximately four-times daily for 3 min each. Cuttings were maintained for approximately 3 weeks, after which they were transferred to 37.9 L plastic containers (Rubbermaid Inc. Wooster, OH, USA) filled with 28 L of well water. Water was analyzed for nutrient concentrations prior to the experiment (**Supplemental Table 1**). A nutrient solution was added to the plastic containers using a complete hydroponic fertilizer (5N-4.8P-21.6K, Peters Professional Hydroponic Special; ICL, St. Louis, MO, USA) and calcium nitrate (14N-0P-0K,17Ca; Calcium + Micros, General Hydroponics, Santa Rosa, CA, USA) dissolved in the well water (**Supplemental Table 1**).

Four rooted cuttings were placed into plastic netted containers (4.7 cm W x 5.1 cm D) spaced equidistant (24.3 cm apart) through the lid of the plastic tub for a given treatment. There were four replicates for every given treatment. Welded wire mesh frames were attached to each lid to support plants. Plants were grown for 4 weeks in the base nutrient solution (**Supplemental Table 1**). A 15.2 cm aquarium air stone attached to an air pump (Active Aqua; Hydrofarm, Petaluma, CA, USA) was added to each plastic container to aerate the solution throughout the experiment. Container volume was maintained by adding well water every 2 d. Four weeks after transplant, nutrient solutions were replaced completely and Cd treatments added using 3CdSO_4_·8H_2_O, to achieve 0, 2.5, 10 and 25 mg·L^-1^ Cd. Cadmium concentrations were chosen based on the results of Huang et al. (2019), who evaluated hemp exposure to Cd in a hydroponic system. Nutrient solutions were maintained to a constant volume by adding water every 2 d and were completely replaced every 2 weeks for the next 10 weeks, except for the 25 mg·L^-1^ Cd tubs, which were harvested after 6 weeks of Cd exposure due to plant senescence caused by Cd toxicity. Electrical conductivity (EC) and pH of the solutions were measured at the start and mid-point of each 2-week period. Solution pH was adjusted to 5.5 when necessary (pH down; General Hydroponics, Santa Rosa, CA, USA). Supplemental light (104 µmol·m^-^²·s^-1^) was used to provide 18/6 light/dark hours for 6 weeks of vegetative growth after planting. Supplemental lights were turned off to allow for flower induction for the remaining 8 weeks of production. Nutrient solutions were sampled at the beginning and end of each 2-week cycle using 20 mL scintillation vials (HDPE; Thermo-Fisher Scientific, Waltham, MS, USA), and stored at -4°C until analysis.

Temperature and relative humidity (RH) of the greenhouse were monitored at canopy height hourly (VP4; Meter Group Inc., Pullman WA, USA) and averaged 19.4 ± 3.6°C and 66% ± 14.0 RH, and 22.1 ± 4.9°C and 63.8 ± 17.8% RH for experiments 1 and 2, respectively. Photosynthetic active radiation was also monitored hourly throughout the experiment (QSO-S; Meter Group Inc.) and the average daily light integral (DLI) was 30.9 ± 18.4 mol·m^-2^·d^-1^ in the first experiment and 47.8 ± 19.7 mol·m^-2^·d^-1^in the second experiment.

### 2.2 Mineral analysis

Samples of the hydroponic solutions were filtered using a 0.45 *µ*M PTFE membrane and acidified using 2% high purity nitric acid (HNO_3_) prior to analysis. At harvest [68 d after Cd treatments, (DAT)], root, stem, leaf, and flower material were collected for metal analysis. Samples were taken prior to air drying whole plants for plant biomass quantification. For each replicate, sub-samples were taken from 3 plants per replicate and combined into a composite sample. In total, four replicates per treatment were used for determination of mineral composition. For roots, approximately 50 g of fresh material was collected from each replicate and triple washed with deionized water prior to drying. For leaves, ten of the youngest fully expanded leaves were collected from the top one-third of each plant (main stem and lateral branches) and rinsed with deionized water. Stem samples of approximately 50 g were collected from the bottom two-thirds of the main stem from each replicate. Approximately 20 g of fresh flower material was sampled from each replicate from the main stem and top one-third of plants.

Plant tissue samples were placed in a forced air oven to set at 55°C for 72 h until a constant weight was achieved. Dried plant material was then ground in a Wiley mill (Thomas Scientific, Swedesboro, NJ, USA) and passed through a 20-mesh screen. The samples were digested following EPA Method 3052 (USEPA Method 3052, 1995) as follows: 0.5 g samples were weighed and placed in fluorocarbon polymer microwave vessels. Then 10 mL of concentrated nitric acid was added to each vessel and sealed, placed in a microwave digester (Mars 6 Microwave; CEM Corp., Matthews, NC, USA), and heated to 200°C for 30 minutes. Digests (solutions) were then transferred quantitatively into volumetric flasks and brought to 100 mL volume with deionized water prior to analysis.

Nutrient solutions and plant tissue digestions were analyzed for multiple elements (P, K, S, Ca, Mg, Fe, Mn, Al, B, Cu, Zn, Ni, and Cd) following EPA Method 200.8 (Creed et al., 1994) by Inductively Coupled Plasma - Optical Emission Spectroscopy (Spectro Arcos FHS16; Spectro Amertek USA, Wilmington, MA, USA). The instrument detection limit for Cd was 0.005 mg·L^-1^. Calibration standards utilized in this analysis were from a certified source (Inorganic Ventures, Christiansburg, VA, lot number: N2-MEB667614). Independent laboratory performance checks were also run with acceptable deviations for recoveries set at 100 ± 5.0%.

### 2.3 Plant biomass and photosynthetic measurements

Plant height and leaf, flower, stem, and root biomass were determined at harvest. Whole plant samples (three per replicate) were air dried at ambient temperatures inside the greenhouse for 2 weeks and then separated into roots, stems, and combined flower and leaf biomass. To adjust for any remaining moisture content, subsamples were taken from the air-dried materials and further dried in a forced air oven set at 55°C for 48 hours until a constant weight was achieved. The dry weights of the whole plant samples were then normalized based on subsample moisture content.

The maximum quantum yield of photosystem II (F_v_/F_m_) was measured during darkness with a fluorometer (Mini-Pam; Walz Company, Effeltrich, Germany) and chlorophyll content index (CCI) was measured at mid-morning with a handheld meter (MC-100; Apogee Instruments, Logan, UT, USA) at 14 and 45 DAT. Measurements were taken on three plants per replicate, on the youngest fully mature leaf on each of the plants.

### 2.4 Cannabinoid analysis

Approximately 25 g of fresh flower tissue obtained from inflorescences located on the top one-third of the plants were sampled from each of the four replicates during week 8 of flowering (68 DAT) and dried separately from other samples for cannabinoid analysis. Four replicates per treatment were used for determination of cannabinoids. Flower material was spread evenly on a perforated baking sheet and dried to approximately 15% moisture content in a walk-in cooler with a temperature set point of 13°C and 55% relative humidity for 14 d. Relative humidity was maintained using a dehumidifier. After 14 d, flower material was hand trimmed to remove leaves and sealed in a metalized resealable food bag (Uline, Braselton, GA) and stored at -4°C for cannabinoid analysis.

The acidic and neutral (decarboxylated) forms of the cannabinoids, THC and CBD, were determined in dried flower material according to the method of Storm et al. (2020) by a commercial laboratory using high performance liquid chromatography and a diode array detector set to 230 nm (SJ Labs & Analytics, Macon, GA). In brief, a 200 mg sample of homogenized dried flower material was extracted with 20 mL of methanol in a 50 mL centrifuge tube. Tubes were vortexed for 10 min, centrifuged at 5000 rpm for 5 min and a 50 µL aliquot of supernatant diluted with 950 µL of methanol and filtered through a 0.45 µm regenerated cellulose syringe filter (4mm Captiva; Agilent, Santa Clara, CA, USA). Analysis was done using high performance liquid chromatography (1220 Infinity II LC; Agilent) with a variable wavelength diode array detector (Agilent). Ten µL of the methanol extract was injected into a 3.0 x 50 mm, 2.7 µm column liquid chromatography column (Infinity Lab Poroshell 120 EC-C18; Agilent). The flow rate was 1.0 mL·min^-1^ for the run. Eluents were A) 0.1% aqueous formic acid B) 0.1% formic acid in methanol. A gradient run was programmed as follows: 40% A and 60% B for 1 min, 40% to 23% A and 60% to 77% B for the next 7 min, then 5% A and 95% B for 2 min. Total cannabinoid concentrations were calculated by the following formula: total cannabinoid = neutral + (acidic form x 0.877). Percentage dry matter for all samples was recorded and results reported on a dry weight basis.

### 2.5 Identification of heavy metal transporter and cannabinoid biosynthesis genes

**Figure 1:**
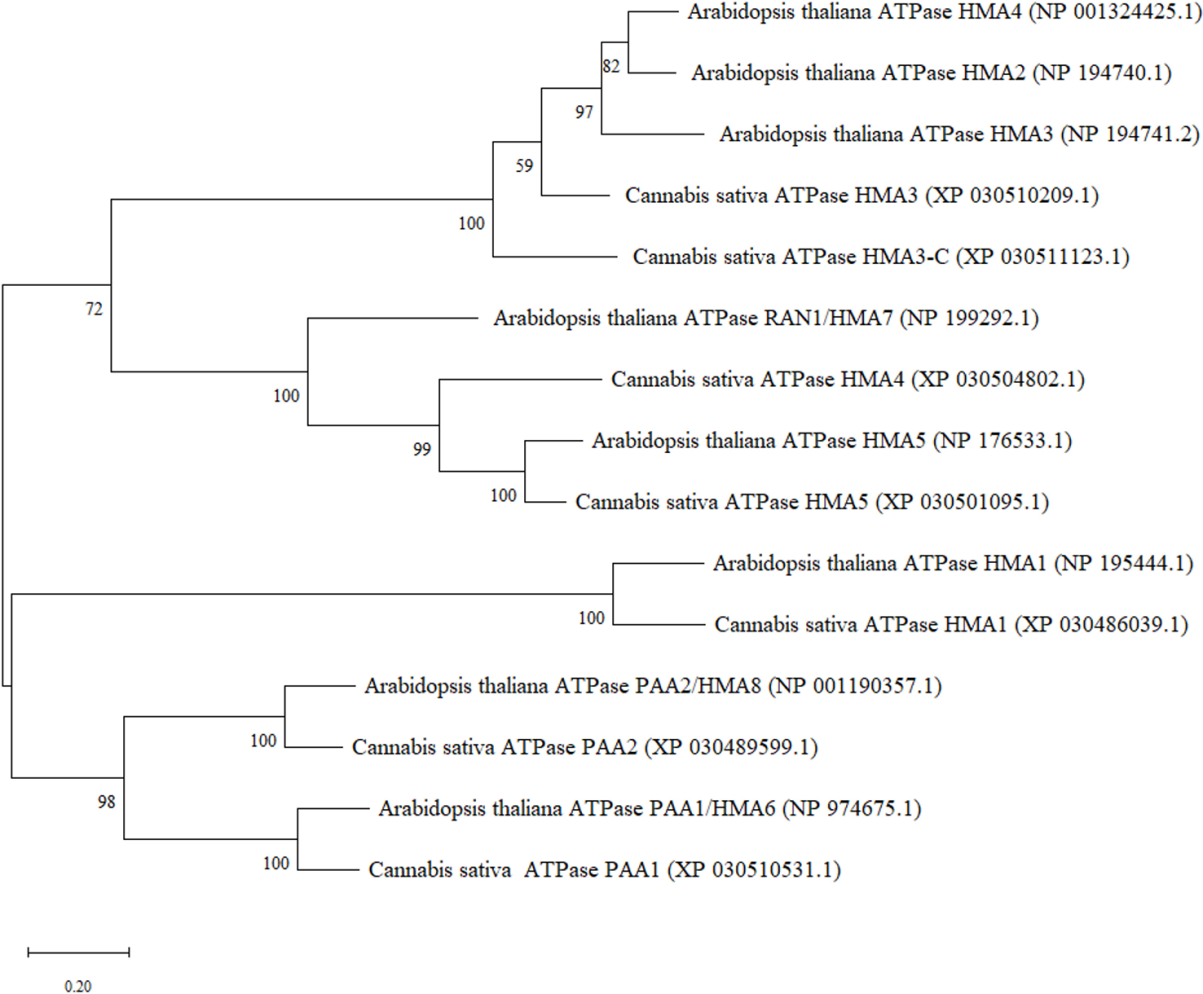
Maximum Likelihood (ML) phylogenetic tree of hemp (*Cannabis sativa* L.) heavy metal ATPases and their closest orthologues in *A. thaliana*.

In order to identify heavy metal transporters in *C. sativa*, orthologue sequences from Arabidopsis P1B-type ATPase gene family containing eight genes were used (**Supplemental Table 2**). Arabidopsis HMA protein sequences from National Center for Biotechnology Information (NCBI) were used to perform blast analysis using blastp function in NCBI against *C. sativa*. Reciprocal blast from *C. sativa* were performed to confirm the identity of the protein. Seven transporter genes were retrieved in *C. sativa*. All the accession numbers for protein sequences have been provided (**Figure 1**). Two cannabinoid synthase genes sequences, *TETRAHYDROCANNABINOLIC ACID SYNTHASE* (XM_030625046.1) and *CANNABIDIOLIC ACID SYNTHASE* (XM_030624886.1), were retrieved from NCBI from accession numbers reported previously (Husain et al., 2019). Further, two *C. sativa* reference genes sequences, *ELONGATION FACTOR 1 ALPHA*/*HBS1-LIKE PROTEIN* (XM_030654944.1) and *UBIQUITIN-PROTEIN LIGASE/UBIQUITIN DOMAIN-CONTAINING PROTEIN DSK2B* (XM_030630092.1), were used to normalize the data and ensure validity of results. These reference genes were found to be stable for quantitative RT-PCR in *Cannabis sativa* L. (Guo et al., 2018). The accession numbers provided in this study were used to retrieve more updated sequences from the *C. sativa* Updated Annotation Release 100 with the accession numbers provided above.

### 2.6 Gene expression analysis

The transcript expression of seven heavy metal transporter genes and two cannabinoid biosynthetic genes identified from *C. sativa* were assessed in root, leaf, and flower tissues at different physiological time points: 2 DAT (vegetative stage), 45 DAT (4 weeks of flowering stage), and 68 DAT (8 weeks of flowering stage). Due to similar trends in plant responses between the two experiments, plant material from experiment 1 was analyzed for gene expression. Approximately 15 g of root, leaf, and flower tissue were collected at 2, 45, and 68 DAT at mid-morning, flash frozen in liquid nitrogen, and stored at –80°C until RNA extraction. Since the plant was at the juvenile stage at 2 DAT, one whole plant per replicate was harvested. For the remaining time points, sub-samples of roots, flowers, and young fully expanded leaves (from the top one-third of each plant) were collected from three plants per replicate and combined. Four replicates per treatment were used for gene expression analysis. For extraction, frozen plant tissues were ground into a fine powder by hand using a mortar and pestle and liquid nitrogen. Total RNA was extracted from 100 mg samples following TRIzol^®^ methodology for leaf tissue and utilizing an E.Z.N.A.^®^ Plant RNA Kit (Omega Bio-Tek, Norcross, GA, USA) for root and flower tissues, following the manufacturers’ recommendation. RNA concentration, 260/280 absorbance ratios, and quality were assessed utilizing Nanodrop (Nanodrop 8000 Spectrophotometer, Thermo Fisher Scientific) and agarose gel electrophoresis.

Following RNA extraction, DNAse treatment was performed at 37°C for 34 minutes in a thermocycler (Mastercycler^®^ X50s Eppendorf SE, Hamburg, Germany). Subsequently, cDNA was synthesized using ImPromII Reverse Transcriptase (Mayorga-Gómez and Nambeesan, 2020). Quantitative RT-PCR reactions were performed in a 96-well plate using PowerUP SYBR Green PCR Master Mix (Applied Biosystems, Foster City, CA, USA), diluted cDNA, and 0.2 μM concentration of forward (Fw) and reverse (Rv) primers using AriaMx Real-time PCR System (Agilent, Santa Clara, CA, USA). Reaction conditions were as follows: 2 min at 50°C, 5 min at 95°C, 40 amplification cycles of 30 s at 95°C followed by 1 min at 60°C, and one final cycle for dissociation curve analysis of 1 min at 95°C followed by 30 s at 55°C followed by 30 s at 95°C. Each reaction was performed at least in triplicates and PCR reaction efficiency was determined by LinRegPCR (v. 11.0). Primers were designed manually by importing sequences into MEGA software (v. 11.0, Tamura et al., 2021) and primer quality was verified using the OligoAnalyzer tool (Integrated DNA Technologies, Coralville, IA, USA). Primer sequences and accession numbers for the genes analyzed can be found in **Supplemental Table 2**.

### 2.7 Phylogenetic analyses

Protein sequences of all the HMA genes in *A. thaliana* and *C. sativa* were used to construct a phylogenetic tree using MEGA software (v. 11.0, Tamura et al., 2021). The evolutionary history was inferred by using the Maximum Likelihood method and JTT matrix-based model. The tree with the highest log likelihood (-11628.46) was used for the construction of the tree. Multiple-sequence alignment was performed with MUSCLE. Phylogeny clusters reliability was tested with 1000 replicates (bootstrap analysis). Closest orthologue sequences were selected by blastp (query cover > 80% and identity > 60%) in the NCBI database.

### 2.8 Statistical and correlational analysis

Experiments followed a complete randomized block design. Statistical analysis was conducted using JMP^©^ Pro 15 (SAS, Cary NC, USA). Data were subjected to a one-way ANOVA procedure with Tukey’s honest significant difference (HSD) test (*P*<0.05) conducted for mean separation when appropriate. Tissue Cd concentrations were log-transformed to ensure equal variance prior to statistical analysis. Non-transformed data are presented. Correlations between Cd concentrations and gene transcript abundance in roots were determined using Spearman correlations using JMP^©^ Pro 15 and p-values were adjusted using the false discovery rate (FDR) method. Heatmaps were created using RStudio (v. 2022.07.0) after transforming the data using Log2 (fold-change + 0.001), where 0.001 was added to account for the genes that showed no expression.

## 3 Results and discussion

### 3.1 Plant growth and yield

**Figure 2:**
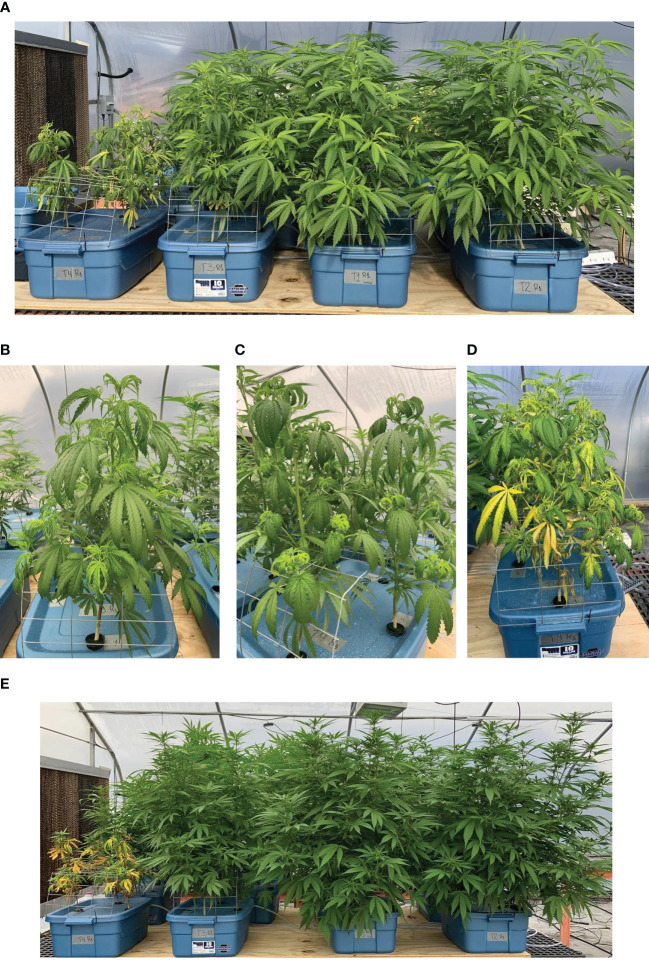
Hemp (*Cannabis sativa* L.) ‘Purple Tiger’ grown hydroponically. From left to right: plants under 25, 10, 0, and 2.5 mg·L^-1^ Cd exposed to treatments for 11 d **(A)**. Plants exposed to 10 and 25 mg·L^-1^ Cd for 2 d [**(B, C)**, respectively]. Plants exposed to 25 mg·L^-1^ Cd for 11 d **(D)**. From left to right: plants under 25, 10, 0, and 2.5 mg·L^-1^ Cd exposed to treatments for 30 d **(E)**.

Plants exposed to 0 and 2.5 mg·L^-1^ Cd grew similarly and displayed no visual symptoms of Cd toxicity throughout the experiment (**Figures 2A, E**). Plants exposed to 10 mg·L^-1^ Cd exhibited visual symptoms of Cd toxicity within 2 d of exposure (2 DAT), such as leaf curling and mild chlorosis, however symptoms decreased as plants continued to grow (**Figure 2B**). Plants exposed to the highest level of Cd (25 mg·L^-1^) exhibited Cd toxicity symptoms within 2 d of exposure (**Figure 2C**). However, unlike plants exposed to 10 mg·L^-1^ Cd, symptoms in plants exposed to 25 mg·L^-1^ Cd continued to worsen over time, with leaves and roots eventually becoming necrotic (**Figures 2C–E**). Plants exposed to 0, 2.5, and 10 mg·L^-1^ Cd were harvested at week 8 of flowering (68 DAT); however, plants exposed to 25 mg·L^-1^ Cd senesced prematurely and were harvested prior to flowering (45 DAT).

### 3.2 Plant biomass

**Figure 3:**
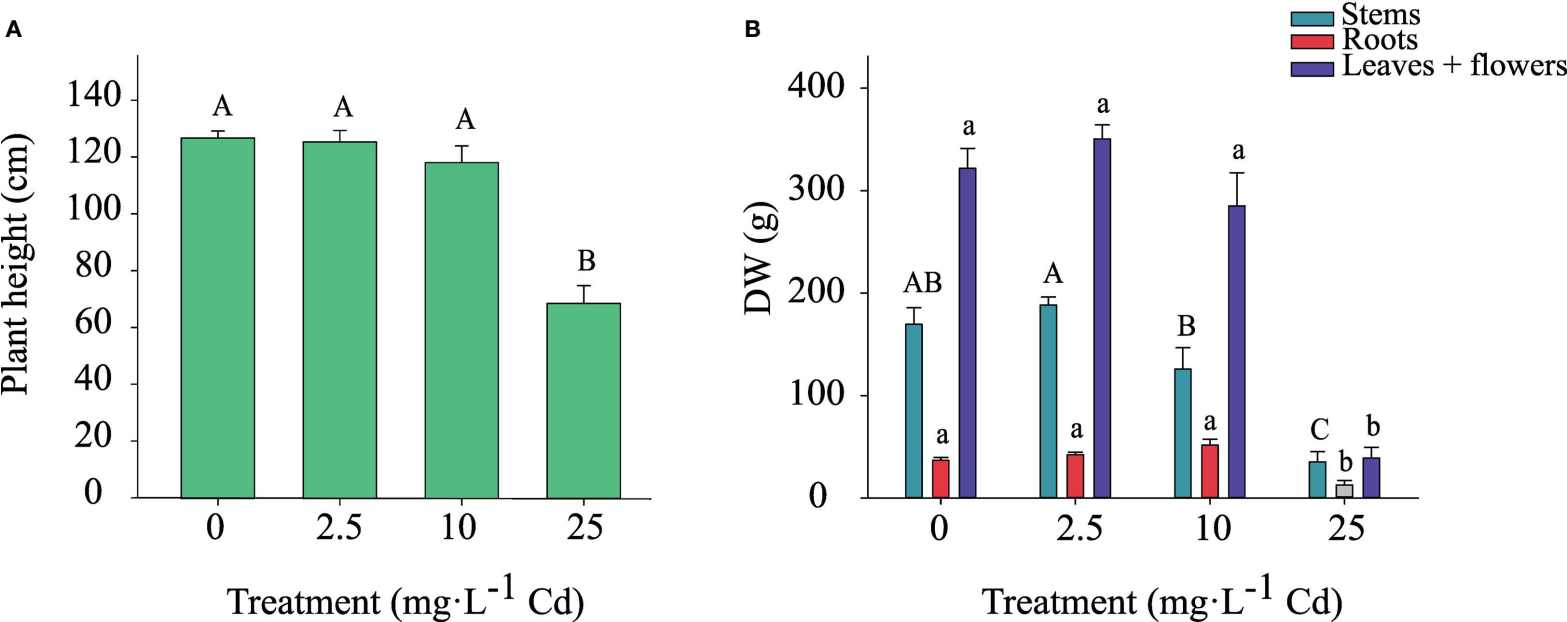
Shoot height ± SE at harvest **(A)**. Dry weight of stems, roots, and flowers + leaves at harvest ± SE **(B)**. Bars associated with the same letter(s) are not significantly different at *P ≤* 0.05 according to Tukey’s HSD all pairwise comparison test.

Shoot height and dry weight of stems, roots, and flower + leaves (classified as ‘biomass’ in industry standards) responded similarly to Cd exposure. Therefore, data from the two experiments were pooled. Shoot height was not affected by Cd for plants exposed to 0, 2.5, and 10 mg·L^-1^ Cd; however, shoot height was reduced in plants exposed to 25 mg·L^-1^ Cd (**Figure 3A**). Dry weight of stems, roots, and flowers + leaves were also greater in the 0, 2.5, and 10 mg·L^-1^ Cd treatments compared to 25 mg·L^-1^ Cd (**Figure 3B**). Compared to the 2.5 mg·L^-1^ Cd treatment, the 10 mg·L^-1^ Cd treatment displayed numerically lower stem biomass, however it was not significantly different from 0 mg·L^-1^ Cd treatment. Root dry weight was lower in the 25 mg·L^-1^ Cd treatment compared to all other treatments. There were no differences in flower and leaf biomass between the 0, 2.5, and 10 mg·L^-1^ Cd treatments, while plants grown with 25 mg·L^-1^ Cd began to senesce prior to full flower development resulting in low production of flower + leaf biomass. Stems, root, and flower + leaf biomass were reduced in the 25 mg·L^-1^ Cd treatment to a greater extent than shoot height, which is congruent with prior results suggesting that plant biomass is a more appropriate reference for analyzing Cd toxicity than shoot growth (Shi & Cai, 2009; Shi et al., 2012). Current results agree with prior studies, suggesting that diminished biomass is a common indicator of Cd toxicity in hemp plants and that Cd toxicity symptoms include leaf chlorosis, leaf curling, and growth inhibition (Linger et al., 2005; Shi & Cai, 2009; Shi et al., 2012; Luyckx et al., 2021). Although plants exposed to 10 mg·L^-1^ Cd visually displayed less branching and thinner stems compared to 0 and 2.5 mg·L^-1^ Cd treatments (personal observation), this was not reflected in the overall root, and flower + leaf biomass. These data also suggest that hemp ‘Purple Tiger’ can tolerate exposure to 10 mg·L^-1^ Cd without alterations in plant biomass.

### 3.3 Cadmium concentration in plant tissues

Analysis of Cd in nutrient solutions at the beginning and end of each 14-d cycle indicates that Cd treatments were applied at intended levels and that Cd was not completely depleted during the growing cycle (**Table 1**). With increasing concentrations of Cd in the nutrient solution, plant uptake of Cd increased, particularly during the first 28 d of Cd exposure. However, after 28 d of exposure, Cd uptake declined in the 25 mg·L^-1^ Cd treatment. This was concurrent with a decline in plant health. In the 10 mg·L^-1^ Cd treatment, Cd uptake rate increased between 28 and 56 d of exposure but declined after 56 d of exposure. These data suggest a continued uptake of Cd during plant growth and development phase including flowering. However, considering that plants exhibited growth during this entire period, increased uptake may be due to increase in plant biomass.

**Table 1:** Mean concentrations of cadmium (Cd) in nutrient solutions measured at the beginning and end of each 14-d cycle and daily uptake.

		mg·L^-1^ Cd	µmol Cd·d^-1^	
Days after treatment	Treatment	Cd initial	Cd final	Average uptake
0-14	0	0.00 ± 0.00	0.00 ± 0.00	0.00 ± 0.00 C^i^
	2.5	2.48 ± 0.03	1.96 ± 0.009	9.22 ± 0.31 C
	10	10.00 ± 0.13	7.77 ± 0.22	39.69 ± 3.85 B
	25	28.33 ± 0.37	21.27 ± 0.36	125.58 ± 20.41 A
14-28	0	0.00 ± 0.00	0.00 ± 0.00	0.00 ± 0.00 B
	2.5	2.49 ± 0.02	1.23 ± 0.21	22.44 ± 1.97 B
	10	9.89 ± 0.18	7.17 ± 0.48	48.37 ± 6.22 AB
	25	28.05 ± 0.33	22.52 ± 1.26	98.32 ± 81.69 A
28-42	0	0.00 ± 0.00	0.00 ± 0.00	0.00 ± 0.00 B
	2.5	2.33 ± 0.03	1.20 ± 0.19	20.04 ± 3.18 B
	10	10.96 ± 0.32	4.99 ± 0.57	106.15 ± 45.53 A
	25	25.32 ± 0.78	23.72 ± 0.56	29.02 ± 21.55 B
42-56	0	0.00 ± 0.00	0.00 ± 0.00	0.00 ± 0.00 B
	2.5	2.59 ± 0.03	2.06 ± 0.08	7.87 ± 11.13 B
	10	11.41 ± 0.14	6.41 ± 0.34	88.85 ± 4.50 A
56-68	0	0.00 ± 0.00	0.00 ± 0.00	0.00 ± 0.00 B
	2.5	2.62 ± 0.13	2.07 ± 0.07	9.86 ± 3.83 B
	10	10.03 ± 0.33	7.14 ± 0.07	51.33 ± 43.90 A

i Values followed by the same uppercase letter(s) within a column are not significantly different for each treatment cycle according to Tukey’s Honest Significant Difference test (P<0.05).

Values are averages ± SE of two experiments each with four replications per treatment.

There was a significant Cd by experiment interaction for Cd concentrations in plant tissue; therefore, Cd accumulation data are presented separately for each experiment. Although Cd concentrations in the different tissues were generally higher in experiment 1, overall Cd accumulation trends were similar in both experiments (**Table 2**). Cadmium concentrations were greatest in root tissue, ranging from 2.5 to 12,663 mg·kg^-1^ in the 0 and 25 mg·L^-1^ Cd treatments, respectively. With increasing Cd concentrations in the solution, there was an increase in Cd concentration in root tissue in all Cd treatments compared to the control treatment. Cadmium concentration in the roots was approximately 150-times greater than in the leaves for the 2.5 mg·L^-1^ Cd treatment, indicating that hemp preferentially sequesters Cd in roots, as reported in previous studies (Citterio et al., 2003; Angelova et al., 2004; Linger et al., 2005; Shi & Cai, 2009; Shi et al., 2012; Luyckx et al., 2021).

**Table 2:** Average cadmium (Cd) concentration in hemp (*Cannabis sativa* L.) ‘Purple Tiger’ plant tissue on a dry weight (dw) basis.

Treatment (mg·L^-1^ Cd)	Flowers	Roots	Leaves	Stems
Cd concentration (mg·kg^-1^)				
Experiment 1				
0	ND^iii^ C^i^ b^ii^	2.5 C a	0.2 D b	ND D b
2.5	8.7 B d	1627.9 B a	11.2 C c	15.3 C b
10	66.2 A c	6146.4 A a	176.3 B b	147.1 B bc
25	NA^iiii^	12663.5 A a	589.4 A c	1344.4 A b
Experiment 2				
0	ND C b	4.7 C a	ND D b	ND D b
2.5	7.6 B c	1982.6 B a	13.2 C b	5.1 C c
10	25.5 A bc	4781.7 AB a	38.9 B b	20.7 B c
25	NA	7436.3 A a	381.9 A b	353.3 A b

i Values followed by the same uppercase letter(s) within a column are not significantly different according to Tukey’s Honest Significant Difference test (P<0.05).

^ii^Values followed by the same lowercase letter within Cd treatment indicate no significant differences among plant tissues according to Tukey’s Honest Significant Difference test (P<0.05).

^iii^ND, not detected.

^iiii^NA, not available for sampling due to inability to form floral tissue.

The 10 mg·L^-1^ Cd treatment had higher Cd concentrations in leaf, flower, and stem tissues compared to 0 and 2.5 mg·L^-1^ Cd treatments. These results suggest that hemp plants subjected to 10 mg·L^-1^ Cd treatment tolerated increased Cd concentrations in multiple tissues without significant alterations in plant biomass. Plants in the 25 mg·L^-1^ Cd treatment exhibited the highest Cd concentrations in leaf and stem tissues in both experiments. Plants exposed to 25 mg·L^-1^ Cd senesced before full flower development was completed; therefore, Cd concentration was not measured in floral tissue in this treatment.

Previously Cd concentrations in stems were reported to be greater than leaves in hemp fiber cultivars (Angelova et al., 2004; Luyckx et al., 2021). In the present study, Cd distribution within flower, leaf, and stem tissues was inconsistent between the two experiments, suggesting that environmental conditions may impact metal concentrations in different plant tissues. Average air temperatures were increased slightly in experiment 2 (22.1 °C) compared to experiment 1 (19.4°C). However, the average DLI increased from 30.9 mol·m^-2^·d^-1^ in experiment 1 to 47.8 mol·m^-2^·d^-1^ in experiment 2 due to increasing day length and light intensities during the second experiment. While the role of light intensities on Cd accumulation has not been thoroughly evaluated, there is evidence that light spectrum can influence transcript abundance of the metal transporter gene *HMA3*, affecting Cd tolerance in cucumber (Guo et al., 2022). Further, *Cannabis* growth and morphology has been documented to respond positively to increasing DLI, which may have resulted in differences in Cd concentrations in the two experiments (Rodriguez-Morrison et al., 2021; Moher et al., 2022)

The cultivar used in current study was selected for cannabinoid production, suggesting that genetics may also affect metal accumulation in plant tissues. Although most previous work has focused on root and stem tissues, a study conducted in metal-contaminated soil reported significant high concentrations of Cd in hemp flowers compared to other plant tissue Angelova et al. (2004).

Our results also suggest that when exposed to significant levels of plant available Cd, such as is the case in a hydroponic production system, Cd can accumulate in floral material in plants with a typical morphology (**Figure 2**). Current limits for Cd in hemp flower range from 0.2 to 0.82 μg/g (California Code of Regulations, 2018; The Maryland Medical Cannabis Commission, 2020; Washington State Liquor and Cannabis Board, 2021). Our data indicate that concentration of Cd in plants exposed to 2.5 mg·L^-1^ Cd was approximately 40 times higher than the legal limit for Cd in hemp flower in several states in the U.S.

### 3.4 Photochemical efficiency

**Figure 4:**
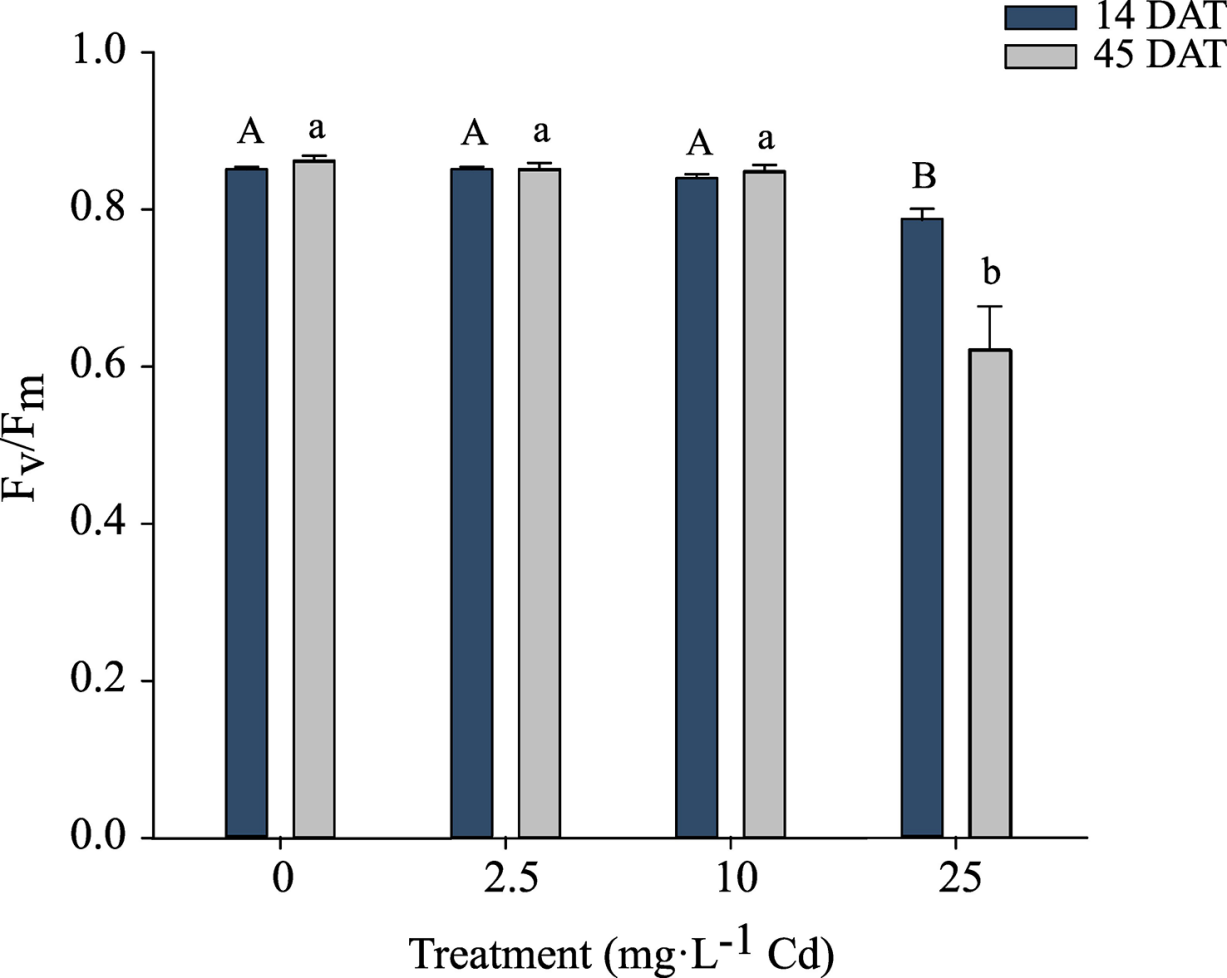
Maximum photochemical yield of Photosystem II (F_v_/F_m_) ± SE in hemp (*Cannabis sativa* L.) ‘Purple Tiger’ 14 and 45 d after treatment (DAT). Bars associated with the same letter(s) are not significantly different at *P ≤* 0.05 according to Tukey’s HSD all pairwise comparison test.

Because there was no significant interaction between Cd treatments and experiments, F_v_/F_m_ data were pooled. There was no impact of Cd on F_v_/F_m_ in plants growing under 0, 2.5, and 10 mg·L^-1^ Cd treatments at 14 or 45 DAT. At 14 and 45 DAT, Cd levels of 25 mg·L^-1^ Cd reduced F_v_/F_m_ to 0.78 and 0.62 respectively, indicating that photosystem II of these plants was damaged in response to the high Cd concentrations (**Figure 4**). Reductions in F_v_/F_m_ indicate photoinhibition damage or other damage to photosystem II in response to environmental stresses (Maxwell and Johnson, 2000).

**Figure 5:**
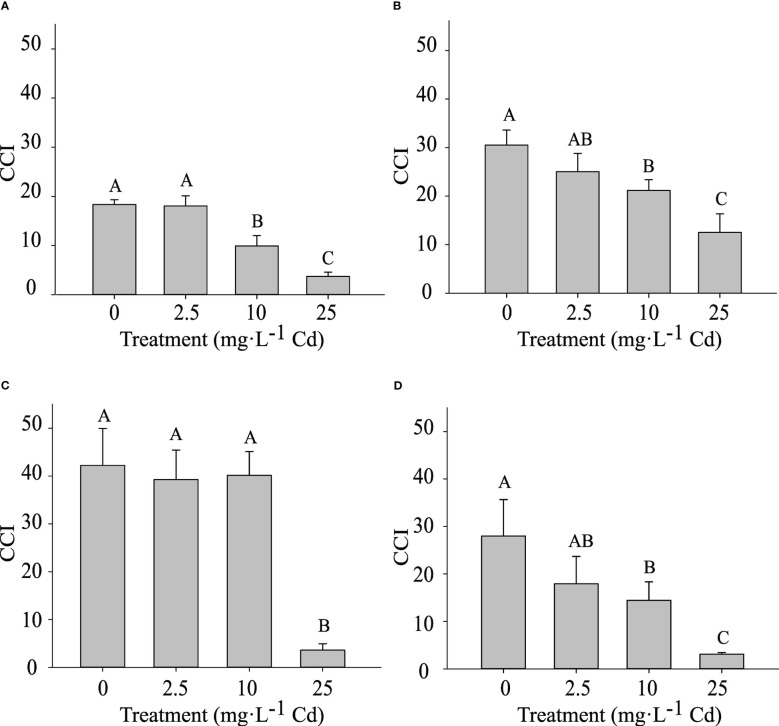
Chlorophyll Content Index (CCI) ± SE in in hemp (*Cannabis sativa* L.) ‘Purple Tiger’ experiment 1 at 14 d after treatment (DAT) **(A)** and 45 DAT **(C)** and CCI in experiment 2 at 14 DAT **(B)** and 45 DAT **(D)**. Bars associated with the same letter(s) are not significantly different at *P ≤* 0.05 according to Tukey’s HSD all pairwise comparison test.

The CCI was measured at 14 and 45 DAT. Despite similar trends, results for the two experiments are shown separately, as there was a significant interaction between experiment and Cd treatment. In experiment 1, plants exposed to 10 and 25 mg·L^-1^ Cd had a lower CCI at 14 DAT when compared to lower Cd treatments (0 and 2.5 mg·L^-1^ Cd) (**Figure 5A**). At 45 DAT, plants exposed to 10 mg·L^-1^ Cd had a similar CCI to the 0 and 2.5 mg·L^-1^ Cd treatments (**Figure 5C**), suggesting that the CCI in the 10 mg·L^-1^ Cd had recovered during the subsequent growth period. However, in experiment 2, the CCI in plants exposed to 10 mg·L^-1^ Cd remained lower than the 0 mg·L^-1^ Cd treatment at 45 DAT. Overall this decrease in CCI at 10 mg·L^-1^ Cd did not affect PSII efficiency (**Figure 4**). This may explain why biomass at 10 mg·L^-1^ Cd was not reduced compared to lower Cd treatments. The CCI in plants exposed to 25 mg·L^-1^ Cd were significantly lower than all the other treatments, throughout both studies, suggesting that the Cd concentrations in leaf tissue in the 25 mg·L^-1^ Cd treatment limited CCI and photosynthetic efficiency. Consistently, leaf chlorosis was observed at this treatment level. The reductions in F_v_/F_m_ at 25 mg·L^-1^ Cd in this study may suggest photoinhibition damage, which has been observed in response to numerous environmental stresses (Maxwell and Johnson, 2000).

Cadmium stress can also trigger oxidative stresses, leading to the accumulation of reactive oxygen species (ROS) (Gillet et al., 2006; Eutropio et al., 2016; Taiz et al., 2015; Singh et al., 2016). Further, metals such as Cd have been reported to replace essential nutrients, disrupting essential reactions in plants (Taiz et al., 2015). These processes can trigger a Cd-induced stress response that can inhibit chlorophyll and carotenoid biosynthesis, disturb chloroplast structure, and ultimately hamper photosynthetic efficiency (Küpper et al., 2002; Mallick and Mohn, 2003; Gillet et al., 2006; Parmar et al., 2013).

### 3.5 Total THC and CBD concentrations

There was an interaction between experiments and Cd treatments for total CBD concentrations, therefore, results from each experiment are presented separately. Total CBD concentrations in the flower material were not impacted by Cd treatments in experiment 1, with average concentrations of 4.94%. However, in experiment 2, CBD concentrations decreased from 9.52% to 7.47% (1.27-fold reduction) in the 0 mg·L^-1^ and 10 mg·L^-1^ Cd treatments, respectively (**Table 3**). Similar to CBD, total THC concentrations were not different among treatments and averaged 0.14% in experiment 1. In experiment 2, there was a reduction from 0.39 to 0.31% (1.25-fold reduction) in the 0 and 10 mg·L^-1^ Cd treatments, respectively.

**Table 3:** Average total CBD and THC in hemp (*Cannabis sativa* L.) Purple Tiger.

	Concentration in flowers (% dw)			
	Experiment 1		Experiment 2	
Treatment (mg·L^-1^ Cd)	Total CBD	Total THC	Total CBD	Total THC
**0**	5.22 A^i^	0.15 A	9.52 A	0.39 A
**2.5**	4.86 A	0.14 A	8.34 AB	0.35 AB
**10**	4.73 A	0.14 A	7.47 B	0.31 B
**25**	NA^ii^	NA	NA	NA

i Values followed by the same uppercase letter(s) within a column are not significantly different according to Tukey’s Honest Significant Difference test (P < 0.05).

^ii^NA, not available for sampling due to inability to form floral tissue.

The legal threshold concentrations of THC imposed by the U.S. for industrial hemp is 0.3%. In experiment 2, these threshold levels were exceeded even under the 0 mg·L^-1^ Cd treatment, suggesting that changes in environmental conditions between experiment 1 and 2 may have influenced total THC content. It is not uncommon for high CBD hemp cultivars, such as Purple Tiger, to have THC concentrations exceeding the 0.3% threshold (Stack et al., 2019; Yang et al., 2020; Coolong et al., 2023). Although variation in cannabinoid concentrations is largely impacted by genetics, it is also understood that there are genetic and environmental interactions that may impact cannabinoid concentrations (Trancoso et al., 2022). Rodriguez-Morrison et al. (2021) and Potter and Duncombe (2011) reported no significant impact of light intensity on cannabinoid concentration in floral tissue, though due to overall increases in yield, total cannabinoid production per plant increased with increasing light intensity. Plants in both experiments were harvested at the same number of days after planting; however, the slightly increased air temperature and increased DLI in experiment 2 may have hastened flower development, which could have led to increased concentrations of cannabinoids in experiment 2 (Yang et al., 2020)

Our results contrast with Husain et al. (2019), who reported total CBD concentrations were greater in the hemp cultivar Fedora 17, grown in mine-land soil containing 0.34 mg·L^-1^ Cd, when compared to plants grown in a commercial substrate containing < 0.25 mg·L^-1^ Cd. However, in agreement with our results, total THC concentrations were higher in plants grown in the commercial substrate compared to Cd-contaminated soil (Husain et al., 2019). Our results do not clearly suggest a link between Cd and concentrations of THC and CBD, particularly since differences were noted only in experiment 2. Further, Cd levels in flower tissue were 2.6-fold lower in experiment 2 than in experiment 1 suggesting a potential interaction between Cd concentration in flowers and environmental factors, leading to a decrease in resources allocated for cannabinoid production (Trancoso et al., 2022).

### 3.6 Transcript abundance of metal transporter and cannabinoid biosynthetic genes

Spatial and temporal expression patterns of genes coding for metal transporters, *CsHMA1*, *CsHMA3, CsHMA3-C, CsHMA4, CsHMA5, CsPAA1*, and *CsPAA2*, were analyzed in roots, leaves, and flowers of plants exposed to Cd treatments at three time points: 2, 45, and 68 (harvest) DAT. In addition, transcript expression of cannabinoid synthase genes, *CsTHCAS* and *CsCBDAS*, were analyzed in leaf and flower tissues. In flowers, none of the transporter and cannabinoid genes analyzed displayed quantifiable expression except for *CsHMA5*. Further, transcript abundance of *CsHMA5* in flowers was also low and not significantly affected by treatments at harvest (**Supplemental Figure 1A**). The transcript abundance of the reference genes in flower tissue was comparable to that in other tissues suggesting optimal RNA integrity. Thus, it is very likely that genes analyzed in this study were not expressed or expressed at very low levels (below the detection limit) in the flower tissue.

**Figure 6:**
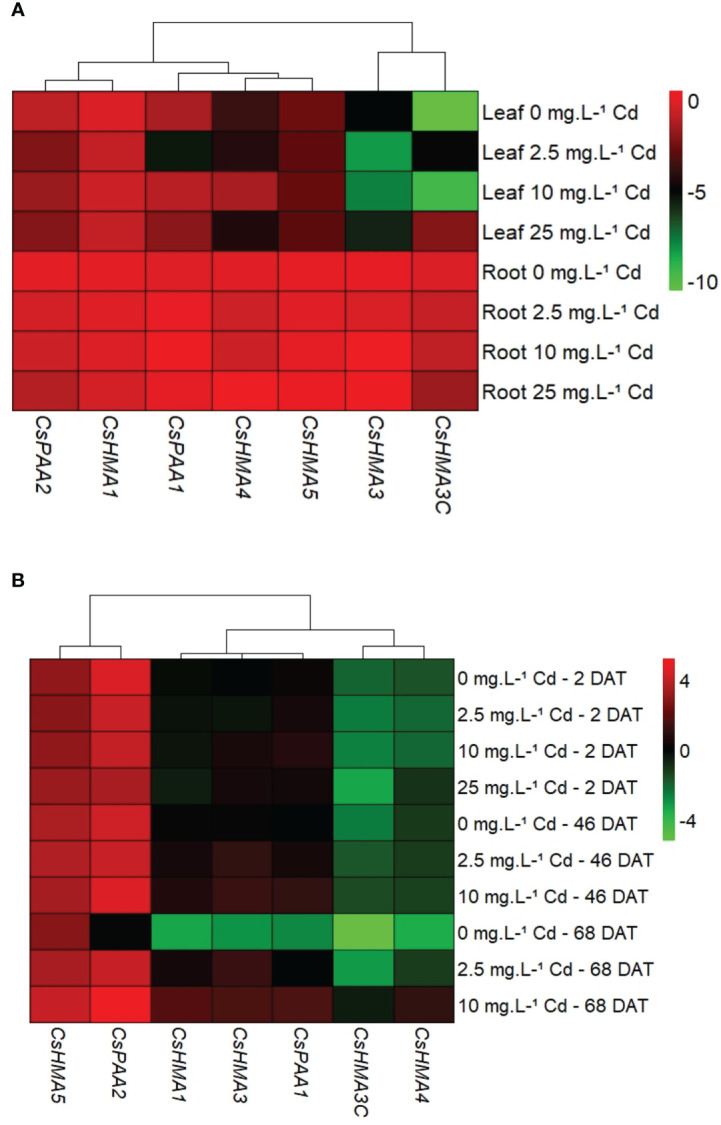
Spatial expression of metal transporter genes at 2 d after treatment (DAT). Transcript abundance of each gene in both tissues was normalized to its expression in roots at 2 DAT **(A)**. Temporal expression of metal transporter genes in root tissues. Transcript abundance of each gene in both tissues was normalized to CsHMA3 expression at 2 DAT **(B)**.

In general, all metal transporters tested had higher transcript abundance in roots when compared to leaves at 2 DAT (**Figure 6A**), and at other time points (data not shown). Transcript abundance of *CsHMA5* in leaves was upregulated by 3-fold in response to the 25 mg·L^-1^ Cd treatment when compared to the control (0 mg·L^-1^ Cd) at 45 DAT (**Supplemental Figure 1B**). However due to Cd toxicity and leaf yellowing, only two replications were analyzed at this stage. Transcript abundance of all seven metal transporters in root tissues was normalized to that of *CsHMA3* exposed to 0 mg Cd·L^-1^ at 2 DAT to identify the most abundantly expressed genes. Among the seven metal transporter genes evaluated, *CsHMA5* and *CsPAA2* were generally highly expressed in roots with *CsHMA1*, *CsHMA3*, and *CsPAA1* showing intermediate expression, and *CsHMA3-C* and *CsHMA4* being least abundant (**Figure 6B**).

**Figure 7:**
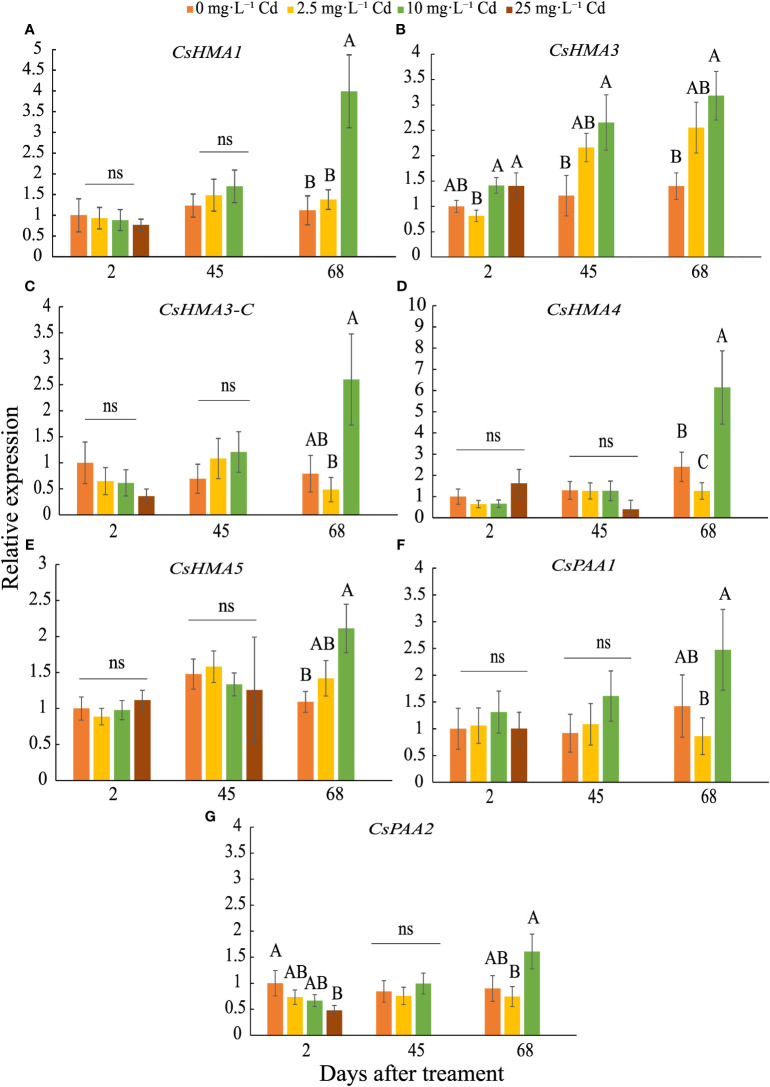
Relative expression ± SE of *CsHMA1* **(A)**, *CsHMA3* **(B)**, *CsHMA3-C* **(C)**, *CsHMA4* **(D)**, *CsHMA5* **(E)**, *CsPAA1* **(F)**, and *CsPAA2* **(G)** in roots of plants exposed to 0, 2.5, 10, and 25 mg.L^-1^ Cd at 2, 45, and 68 d after Cd treatment (DAT). Bars associated with the same letter(s) are not significantly different at *P ≤* 0.05 according to Tukey’s HSD all pairwise comparison test. ns, not significant.

*CsHMA3* was upregulated by 2.7- and 3-fold in plants exposed to 10 mg·L^-1^ Cd at 45 and 68 DAT, respectively (**Figure 7B**). *CsHMA1*, *CsHMA4*, and *CsHMA5* were upregulated by 4-, 6-, and 2-fold, respectively, in plants exposed to 10 mg·L^-1^ Cd at 68 DAT (**Figures 7A, D, E**). The three remaining transporter genes, *CsHMA3-C*, *CsPAA1*, and *CsPAA2* showed a trend of increasing abundance under 10 mg·L^-1^ Cd treatment at 68 DAT, but were not significantly different compared to the control treatment (**Figures 7C, F, G**). Transcript abundance of two transporter genes at harvest, *CsHMA1* and *CsHMA5* were positively correlated to Cd concentrations in roots (**Table 4**).

**Table 4:** Correlation between transcript expression of Cs metal transporter genes and Cd concentration in roots at 68 DAT.

Cs metal transporter gene	Correlation to Cd concentration	*P* value
***CsHMA1***	**0.82**	0.02
***CsHMA3***	0.52	ns
***CsHMA3-C***	0.70	ns
***CsHMA4***	0.52	ns
***CsHMA5***	**0.81**	0.02
***CsPAA1***	0.56	ns
***CsPAA2***	0.68	ns

Significant positive correlations are in bold and ns indicates not significant.

Metal transporters from the heavy metal ATPase (HMA) family have been categorized into two subgroups based on their specificity of metal substrates. These are the Zn-ATPases (Zn^2+^/Cd^2+^/Co^2+^/Pb^2+^ specificity) and Cu-ATPases (Cu^2+^/Ag^2+^ specificity) which may be regulated within certain tissues or subcellular compartments (Takahashi et al., 2012; Wang et al., 2018; Zhang et al., 2018). *A. thaliana* has eight HMA genes of which *AtHMA1-4* belong to the former group whereas *AtHMA5-8* are closely related to the Cu^2+^/Ag^2+^ subclass. Based on the phylogenetic analysis, most similarities were observed between *CsHMA1* and *AtHMA1*, *CsHMA3/CsHMA3-C* and *AtHMA2-4*, *CsHMA4*/*CsHMA5* and *AtHMA7* and *AtHMA5*, *CsPAA1* and *AtHMA6*, and *AtHMA8* and *CsPAA2* (**Figure 1**).

*AtHMA1* has been shown to play role in detoxification of excess Zn and is localized to the chloroplast envelope (Kim et al., 2009). Transcript abundance of *CsHMA1*, most closely related to *AtHMA1*, was upregulated in response to 10 mg·L^-1^ Cd at 68 DAT, suggesting that this gene is transcriptionally regulated during advanced growth stages, perhaps leading to greater plant Cd uptake and accumulation. Previous findings reported on the role of *AtHMA3* and its orthologues on Cd sequestration into root vacuoles (Morel et al., 2009; Miyadate et al., 2011; Ismael et al., 2019). Further, *AtHMA2* and *AtHMA4* were reported to be involved in Cd uptake and translocation, being expressed in tissues adjacent to the root vascular bundle for loading Cd into the xylem (Wong & Cobbett, 2009; Ismael et al., 2019). *CsHMA3* was the only transporter induced during mid and late growth stages at 45 and 68 DAT, and similar to *AtHMA2-4* may be involved in Cd uptake, sequestration into the root vacuoles, and possibly xylem loading. Migocka et al. (2015) reported that in cucumber (*Cucumis sativus*) *HMA3* was upregulated in roots in response to Cd exposure and conferred tolerance to heavy metal detoxification through sequestration in the vacuole. Further, *HMA3* expression was greater in response to Cd exposure compared to *HMA4* in cucumber, though activity of *HMA4* was higher when exposed to elevated levels of Zn, suggesting that *HMA4* may have been more efficient as a Zn^2+^-ATPase compared to a Cd^2+^-ATPase (Migocka et al., 2015).

As noted earlier, *AtHMA5-8* belong to the Cu^2+^/Ag^2+^ ATPase subgroup and functions in Cu transport. *AtHMA5* was predicted to be mainly expressed in roots and involved in Cu^2+^ transport, compartmentalization, and detoxification (Axelsen & Palmgren, 2001; Andres-Colas et al., 2006). Although *CsHMA4* and *CsHMA5* fall within this subclass, our data indicate the upregulation of these transporter genes under long-term Cd stress. Similarly, *Populus tomentosa HMA5* shared similarity with *AtHMA5*, and overexpression of *PtoHMA5* in tobacco plants increased Cd translocation from roots to leaves, suggesting its role in Cd transport (Wang et al., 2018). Similarly, upregulation of a putative Cu-transporting ATPase, *HMA5* was found to be induced by Cd exposure in peanut (Chen et al., 2019) and durum wheat (Aprile et al., 2018).

Overall, the seven transporter genes were expressed in root tissue and potentially involved in cation uptake, including Cd. When Cd levels increased during the course of plant growth under the 10 mg·L^-1^ Cd treatment, *CsHMA1*, *CsHMA3*, *CsHMA4* and *CsHMA5* transcript abundance were upregulated in the roots possibly to allow for increased uptake, sequestration, and translocation of Cd. These transporters may play a role in leaf Cd uptake as well; however, leaf Cd concentrations were substantially lower compared to that in roots and may not elicit an increase in their transcript abundance.

Of the two cannabinoid biosynthetic genes, *CsCBDAS* and *CsTHCAS*, analyzed in leaf tissue, only the expression of *CsTHCAS* was downregulated at harvest in plants exposed to 10 mg·L^-1^ Cd (**Supplemental Figure 2B**). Cannabinoid levels in flower tissue did not change in experiment 1 and transcript abundance of cannabinoid biosynthetic genes in leaf tissues with cannabinoid levels in flower tissue may not be well correlated. Although previous studies have indicated upregulation of *CsCBDAS* in flowers with higher total CBD concentrations in Cd contaminated soil (Husain et al., 2019), other studies have proposed that transcript levels of both *CsCBAS* and *CsTHCAS* are not well correlated with CBD and THC synthesis (Apicella et al., 2022).

## 4 Conclusions

Our findings indicate that *C. sativa* may tolerate Cd exposure of 2.5 and 10 mg·L^-1^ Cd in a hydroponic solution. Concentrations of 25 mg·L^-1^ Cd severely restricted growth, reduced F_v_/F_m_, CCI, and caused premature death. As expected, Cd concentrations were highest in roots, but there was significant accumulation of Cd in leaves, stems, and flowers, with increasing Cd in the nutrient solution. Plant biomass and total CBD and THC in flowers were not significantly different between plants in the control treatment and plants exposed to up to 10 mg·L^-1^ Cd. Transcript abundance of *CsHMA1*, *CsHMA3*, *CsHMA4*, and *CsHMA5* in the roots of *C. sativa* were upregulated in 10 mg·L^-1^ Cd treatments, suggesting that they may be associated with Cd uptake, sequestration in roots and xylem loading. While Cd concentrations that were available to plants used in the present study are greater than what would be encountered on agricultural soils, our results indicate that hemp plants have the potential to accumulate Cd in floral tissue. Therefore, heavy metal testing in *C. sativa* consumer products may be of importance.

## Data availability statement

The raw data supporting the conclusions of this article will be made available by the authors, without undue reservation.

## Author contributions

All authors listed have made a substantial, direct, and intellectual contribution to the work and approved it for publication.
