# Supplementary material for: Cadmium exposure is associated with increased transcript abundance of multiple heavy metal associated transporter genes in roots of hemp (Cannabis sativa L.)
Source: Front Plant Sci. 2023 May 30;14:1183249. doi: 10.3389/fpls.2023.1183249 (PMC10265645; doi:10.3389/fpls.2023.1183249)
Supplement: Supplementary file 1 [file DataSheet_1.docx]

Supplementary Material

**Cadmium Exposure is Associated with Increased Transcript Abundance of Multiple Heavy Metal Associated Transporter Genes in Roots of Hemp (Cannabis sativa L.)** Amanda O. Marabesi^1*^, Savithri U. Nambeesan^1^, Marc W. van Iersel^1^, Jason T. Lessl^2^, and Timothy W. Coolong^1*^

^1^Department of Horticulture, University of Georgia, Athens GA 30602, USA.

^2^Agricultural and Environmental Services Lab, University of Georgia, Athens, GA 30602, USA.

**^*^Correspondence:**
Corresponding Authors
[aom@uga.edu](mailto:aom@uga.edu)
[tcoolong@uga.edu](mailto:tcoolong@uga.edu)

# Supplementary Figures and Tables

## Supplementary Tables

| Supplemental Table 1. Mineral nutrient concentrations (mg·L^-1^) used in this study. | | | | | | | | | | | | |
| --- | --- | --- | --- | --- | --- | --- | --- | --- | --- | --- | --- | --- |
|  | N | P | K | Ca | Mg | B | Cu | Mo | Fe | Mn | Zn |  |
| Well Water | ND^i^ | 0.1 | 2.9 | 10.5 | 1.9 | 1.7 | <0.05 | <0.01 | 0.3 | 0.2 | <0.05 |  |
| Nutrient Solution^ii^ | 150 | 50 | 200 | 125 | 65 | 3 | 0.3 | 0.1 | 4 | 1 | 0.3 |  |
| Total Concentration | 150 | 50.1 | 202.9 | 135.5 | 66.9 | 4.7 | 0.3 | 0.1 | 4.3 | 1.2 | 0.3 |  |
| ^i^ND = not detected. ^ii^Nutrient solution comprised from the following compounds: KNO_3_, K_2_HPO_4_, MgSO_4_, H_3_BO_3_, Cu-EDTA, Fe-EDTA, Mn- EDTA, Na_2_MoO_4_, Zn-EDTA, Ca(NO_3_)_2_•NH_4_NO_3_•10H_2_O, (NH_4_)_2_MoO_4_, Ca-EDTA, and Na_2_B_4_O_7_·10H_2_O. | | | | | | | | | | | | |

| Supplemental Table 2. Primer sequences and accession numbers of metal transporter, cannabinoid biosynthetic, and reference genes. | | | |
| --- | --- | --- | --- |
| Gene name | NCBI reference | Fw sequence | Rv sequence |
| *Cs probable cadmium/zinc-transporting ATPase HMA1* | XM_030630179.1 | GACGGAAGCTCAGAAAGCG | ACCTGTAGAACCGCAACAC |
| *Cs putative inactive cadmium/zinc-transporting ATPase HMA3* | XM_030654349.1 | AGGGAAGTGCTGTAAACCT | CCGAGTTCCCGCTCTTCT |
| *Cs putative inactive cadmium/zinc-transporting ATPase HMA3 Complement* | XM_030655263.1 | GGTCATGGCCTGTGGATTATTAC | GTGAGGTTCCAAATGGAGGT |
| *Cs copper-transporting ATPase HMA4* | XM_030648942.1 | CTTCAAGGTCAGGCTGTGA | CGGGAAACTCCTCAGCTGT |
| *Cs probable copper-transporting ATPase HMA5* | XM_030645235.1 | CAATAGCTGCAGGAGCACTC | GAG CAG CAA ACA ACA CTG AC |
| *Cs copper-transporting ATPase PAA1* | XM_030654671.1 | GTGGAAGAAGCCCAAAGTAGG | AAGGTAGCAGTAGAGAGAGCC |
| *Cs copper-transporting ATPase PAA2* | XM_030633739.1 | TGGTCGTGAAGGAGAAGGC | TCCCCGGATAAGAGCAAAGT |
| *Cs inactive tetrahydrocannabinolic acid synthase* | XM_030625046.1 | CCTTCAAATGTCTCCCATATCCAGG | TGTAGGACAAACCCTCAGC |
| *Cs cannabidiolic acid synthase-like* | XM_030624886.1 | GTCACTCCTTCACATGTCTCTC | GGGACTTGAGATATGTAGGACATG |
| *Cs Elongation factor 1 alpha/HBS1-like protein (reference gene)* | XM_030654944.1 | TTGCACGGATCAGTTTGCG | AATGCCGACCGCTACAGTT |
| *Cs ubiquitin-protein ligase/ubiquitin domain-containing protein DSK2b) (reference gene)* | XM_030630092.1 | TACGAGGAGCAGCTGTAGAT | ATAGAGTGAGGGTGGGAGAAGA |

## Supplementary Figures


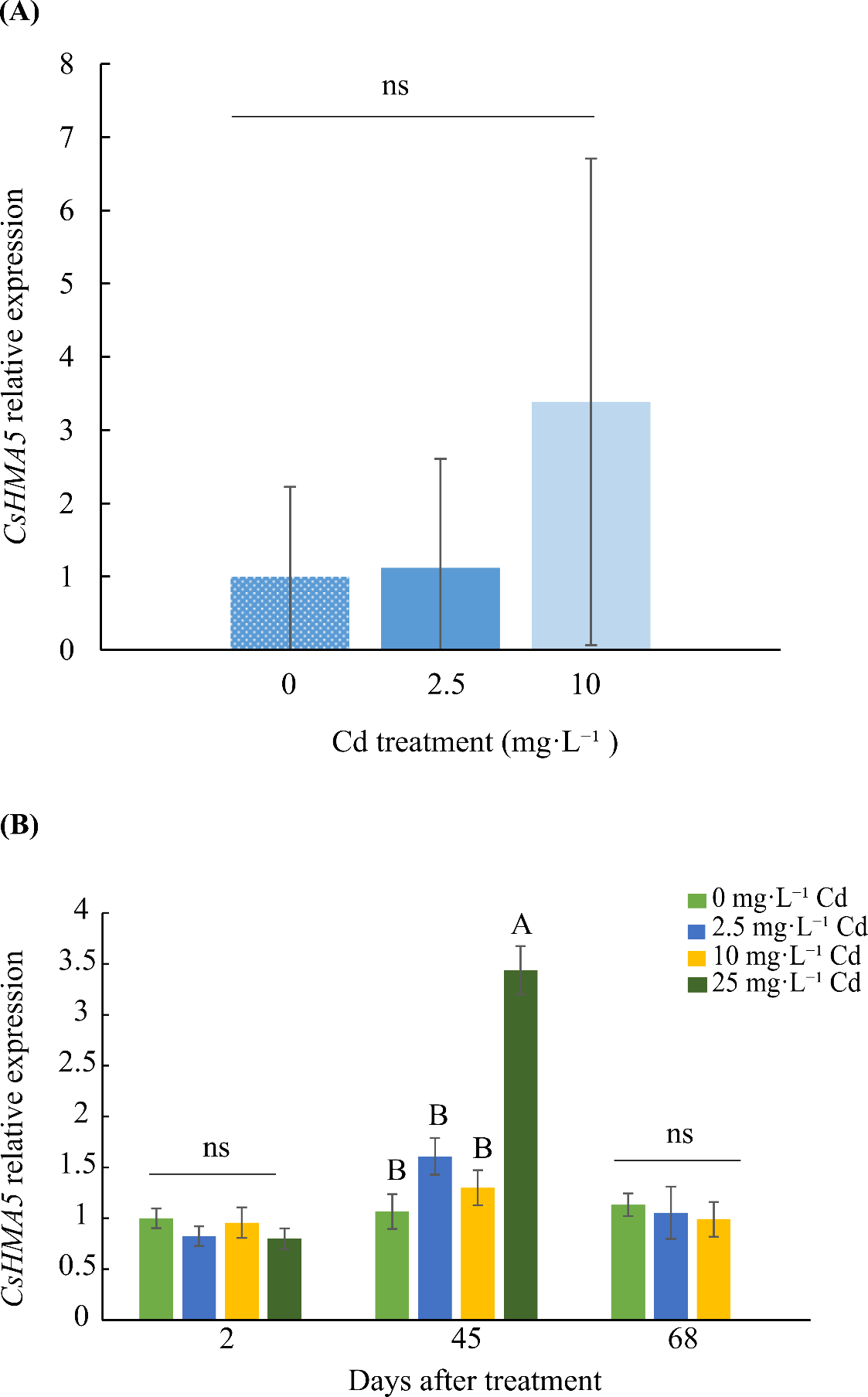


Supplemental Figure 1. Relative expression ± SE of ATPase *CsHMA5* in flower tissue of plants exposed to 0, 2.5, and 10 mg·L^-1^ Cd at 68 d after Cd treatment (DAT) (**A**). Relative expression ± SE of ATPase *CsHMA5* in leaf tissue at 2, 45, and 68 DAT (**B**). Plants were exposed to 0, 2.5, 10, and 25 mg·L^-1^ Cd.Bars associated with the same letter(s) are not significantly different at *P ≤* 0.05 according to Tukey’s HSD all pairwise comparison test.


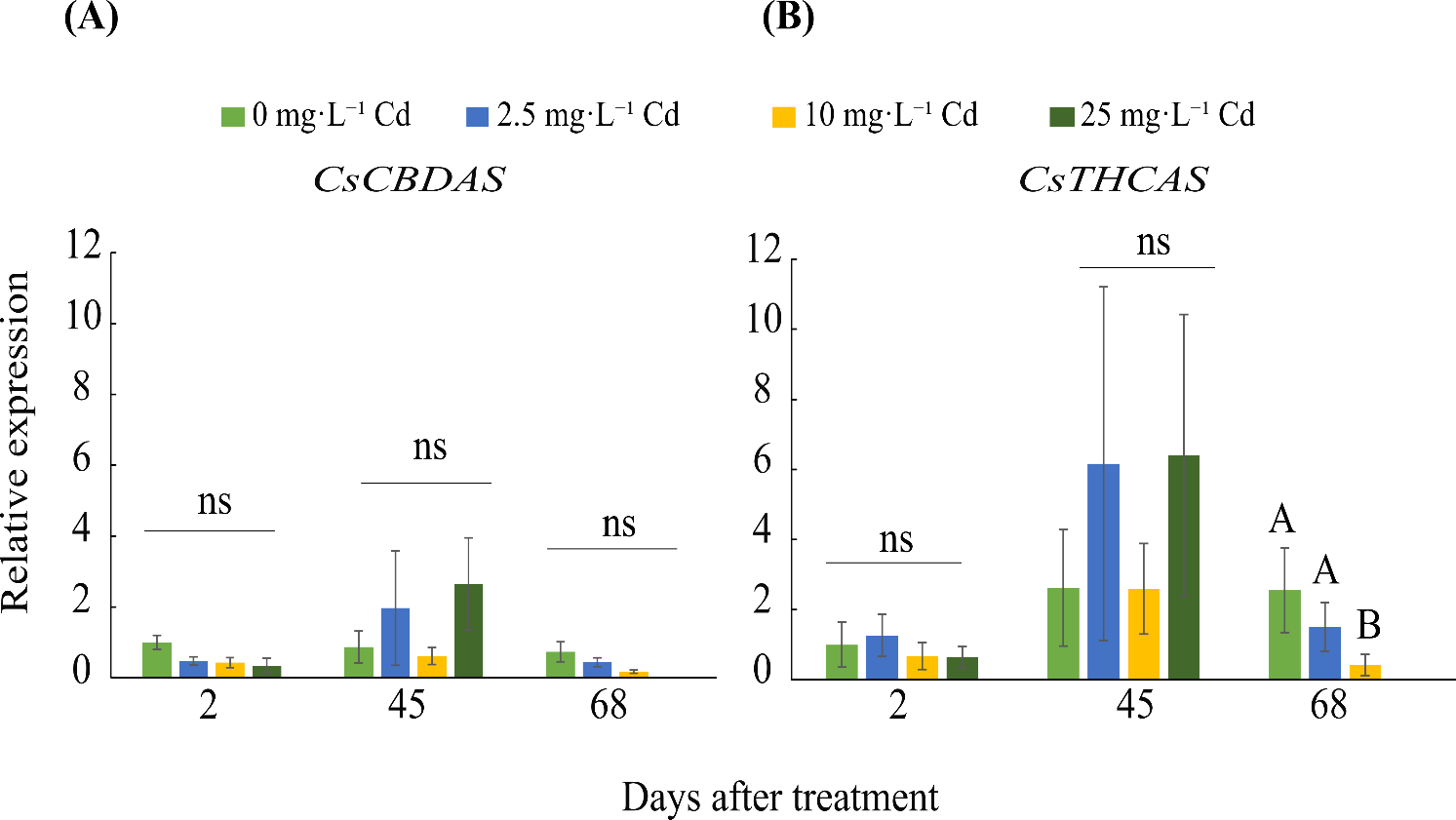


Supplemental Figure 2. Relative expression ± SE of *CsCBDAS* (**A**) and *CsTHCAS* (**B**) in leaf tissue at 2, 45, and 68 days after Cd treatment (DAT). Plants were exposed to 0, 2.5, 10, and 25 mg·L^-1^ Cd. Bars associated with the same letter(s) are not significantly different at *P ≤* 0.05 according to Tukey’s HSD all pairwise comparison test.
